# Error in Byline

**DOI:** 10.1001/jamanetworkopen.2026.4615

**Published:** 2026-03-04

In the Consensus Statement titled “Rationale and Methodological Approach Underlying the Development of the Sequential Organ Failure Assessment (SOFA)–2 Score: A Consensus Statement,”^1^ published October 29, 2025, there was a typographical error in the name of coauthor Hallie C. Prescott, MD, MSc. This article has been corrected.^1^
